# Training Characteristics During Pregnancy and Postpartum in the World’s Most Successful Cross Country Skier

**DOI:** 10.3389/fphys.2018.00595

**Published:** 2018-05-23

**Authors:** Guro S. Solli, Øyvind Sandbakk

**Affiliations:** ^1^Department of Sports Science and Physical Education, Nord University, Bodø, Norway; ^2^Department of Neuromedicine and Movement Science, Centre for Elite Sports Research, Norwegian University of Science and Technology, Trondheim, Norway

**Keywords:** body composition, endurance training, female athlete, parturition, delivery, physiological capacity, strength training

## Abstract

This case-study investigated the training characteristics, physiological capacity, and body composition of the world’s most successful cross country skier during the 40-week pregnancy, and the 61-week postpartum. Training data was systemized by training form (endurance, strength, and speed), intensity [low- (LIT), moderate- (MIT), and high-intensity training (HIT)], and mode (running, cycling, and skiing/roller skiing). The training volume [mean ± standard deviation (median)] during pregnancy was 12.9 ± 7.3(10.0) h/week in the first- (weeks 1–12), 18.3 ± 2.9(18.0) h/week in the second- (weeks 13–28), and 8.8 ± 4.4(9.6) h/week in the third trimester (weeks 29–40). Endurance training time was distributed into 10.9 ± 6.2(9.9), 15.2 ± 2.3(15.6), and 7.6 ± 3.8(7.9) LIT and 0.4 ± 0.5(0.0), 1.3 ± 0.4(1.4), and 0.7 ± 0.6(0.8) h/week MIT during the three trimesters. Only 2.2 h of HIT was performed during the entire pregnancy. During the first two trimesters, the distribution of exercise modes were approximately the same as pre-pregnancy, but the amount of running was reduced during the third trimester. Training volume during the postpartum periods 1–4 was 6.6 ± 3.8(7.1) (PP1; weeks 1–6), 14.1 ± 3.4(14.3) (PP2; weeks 7–12), 10.6 ± 3.8(10.4) (PP3; weeks 13–18), and 13.6 ± 4.1(14.5) h/week (PP4; weeks 19–24), respectively. Training during PP3 and PP4 was interfered with two fractions in the sacrum, leading to decreased amount of running and MIT/HIT, compensated by increased amounts of cycling. Thereafter, training volume progressively approached the pre-pregnancy values, being 18.0 ± 3.9(18.7) h/week during the general preparation- (weeks 25–44), 17.6 ± 4.4(17.3) h/week during the specific preparation- (weeks 45–53), and 16.9 ± 3.5(17.2) h/week during the competition period (CP; weeks 54–61) leading up to the subsequent world championship. The oxygen uptake at the estimated lactate threshold (LT) decreased to 90% of pre-pregnancy values in the second trimester, but remained to ∼100% in PP3. Body weight and fat-% was higher, while lean body mass and bone mineral density was lower after delivery compared to pre-pregnancy. These measurements gradually changed and were back to ∼pre-pregnancy values during CP. This study indicates that high-level cross country skiers can tolerate high training loads during pregnancy. Although the participant had some postpartum setbacks in her training due to fractures in the sacrum, reduced overall training load, followed by a slower progression and utilization of alternative exercise modes, led to a successful return to competitions.

## Introduction

There is an increasing number of women competing in elite sports, and many women want to resume their sporting career after giving birth. Although pregnant elite athletes undergoes the same anatomical, physiological, and biomechanical changes as non-athletes, their training load need to be balanced with the consideration to the health of their own and the fetus. In this context, an international expert committee recently reviewed the literature and provided specific exercise recommendations for elite athletes throughout pregnancy (Bo et al., 2016) and postpartum (Bo et al., 2017b). In general, physical activity and moderate exercise during pregnancy are found to be safe both for the mother and the child (Hellenes et al., 2015; Di Mascio et al., 2016), although 30–60% reduced training volumes during the third trimester has been reported in recreational and competitive runners (Potteiger et al., 1993; Bailey et al., 1998; Tenforde et al., 2015).

Intensive exercise >90% of maternal heart rate, especially during hot and humid conditions, could lead to a hypoxic situation for the fetus (Salvesen et al., 2012; Bo et al., 2016). This is also the case for intensive training at altitudes >1500–2000 m due to decreased fetal arterial oxygen saturation (Entin and Coffin, 2004). However, studies report that fetal hypoxia in connection with exercise is transient (Szymanski and Satin, 2012). Furthermore, the use of heavy strength training during pregnancy is debated. The Valsalva maneuver used during such training induces a rapid elevation of blood- and intra-abdominal pressure, which potentially could be harmful for both the fetus and the pelvic floor support (Harman et al., 1988; Palatini et al., 1989; Hartmann and Bung, 1999). However, recent studies found no deleterious effect on the pelvic floor investigating cross-fit athletes performing heavy lifts (80% of maximum) during pregnancy (Middlekauff et al., 2016). In summary, few studies have investigated the impact of strenuous endurance training and/or heavy strength training during pregnancy in elite endurance athletes (Bo et al., 2017a), and more research is needed to develop more specific recommendations for top athletes.

After giving birth, the athlete has to adjust the training to allow for full recovery and management of the new obligations with a newborn child, including breast-feeding and a potential irregular circadian rhythm (Potteiger et al., 1993). However, training after birth is relatively unexplored. A study, investigating 40 Norwegian elite athletes reported that 38% of these, compared to only 4% of non-athletes, started jogging within the first 6 weeks postpartum (Bo and Backe-Hansen, 2007). A marathon runner reported a training volume as high as 50 km during the initial week postpartum, which was progressively increased to 98 km during week 14 without any major problems (Potteiger et al., 1993). However, drainage of calcium from maternal skeleton during late pregnancy and calcium loss due to breast milk production could potentially lead to increased risk of fragility fractures during the postpartum period (Woodrow et al., 2006; Kovacs, 2011; Sanz-Salvador et al., 2015).

In a recent study, we investigated the training characteristics of the world’s most successful female XC-skier (Solli et al., 2017), with a special focus on five consecutive successful seasons. Immediately after these seasons, the participant got pregnant and born a healthy child before she resumed to training and won four gold medals in the subsequent World Championship. Therefore, the main aim of this case-study was to investigate her training characteristics, physiological capacity and body composition during pregnancy and the year postpartum.

## Methods

### Participant

The participant is the most successful competitor of all time in the winter Olympics, and competed at an international level for 15 years before getting pregnant at the age of 35. The participant is nulliparous from before, and had a singleton pregnancy. The study was evaluated by the Regional Committee for Medical and Health Research Ethics (2017/2070/REK-midt), and approved by the Norwegian Social Science Data Services (NSD). Written informed consent was obtained from the participant for the publication of this case report, which was performed according to the Helsinki declarations.

### Overall Design

This study builds on a previous longitudinal training study (Solli et al., 2017), and investigated the training and test data from conception to delivery, and the 61-week postpartum period until participation in the subsequent World Championship.

### Physiological Testing

The participant underwent physiological testing during the first- and second trimester and regularly after delivery, as specified in **Table 1**. Equipment and procedures for the physiological tests and body composition measurements are previously described (Solli et al., 2017). Because of radiation, no measurements of body composition was performed during pregnancy.

**Table 1:** Physiological characteristics of the world’s most successful cross-country skier during pregnancy and postpartum.

	Pre-pregnancy	During pregnancy			Postpartum period					
		Tri-1	Tri-2	Tri-3	PP1	PP2	PP3	PP4	GP	CP
Age (year)	34.5	35.2	35.4	35.7	35.8	36.0	36.1	36.2	36.5	36.8
Body height (cm)	167	167	167	167	167	167	167	167	167	167
Body mass (kg)	64.0	65.2	67.1	79.0*	69.4	68.1	67.6	68.5	68.3	64.6
Body mass index (kg⋅m^-2^)	22.9	23.4	24.1	28.3*	24.9	24.4	24.2	24.6	24.5	23.2
Lean body mass (kg)	55.0	–	–	–	53.0	–	52.1	54.0	54.5	55.0
Lean upper body mass (kg)	34.5	–	–	–	32.9	–	32.2	33.5	33.9	34.9
Lean lower body mass (kg)	17.6	–	–	–	17.0	–	16.8	17.4	17.5	17.1
Fat %	12.8	–	–	–	20.4	–	18.2	17.9	16.9	11.3
Bone mineral density (g⋅cm^-2^)	1.298	–	–	–	1.199	–	1.154	1.203	1.237	1.250
*Z*-score	0.8	–	–	–	0.0	–	-0.3	0.1	0.3	0.5
*V*O_2_ @LT (ml⋅kg ^-1^⋅min^-1^)	60.8	57.0	54.2	–	–	57.2	59.3	–	–	61.9^#^
*V*O_2_ @LT (L⋅min^-1^)	3.9	3.7	3.6	–	–	3.9	4.0	–	–	4.0^#^

*LT, estimated lactate threshold; VO_2_ @LT oxygen uptake at the lactate threshold (running); Tri-1, first trimester; Tri-2, second trimester; Tri-3, third trimester; PP1, postpartum period one; PP2, postpartum period two; PP3, postpartum period three; PP4, postpartum period four; GP, general preparation period; CP, competition period; ^∗^the highest measured value before delivery; ^#^values measured after the competition period*.

### Monitoring, Registration, and Systematization of Training

All training data was recorded daily by the participant in digital diaries designed by the Norwegian Olympic Federation, which previously has been reported to provide a valid and accurate measurement of the duration and intensity of training by XC-skiers (Sylta et al., 2014). As previously described (Solli et al., 2017), all training data was systematized by training form (endurance, strength, and speed), intensity [low- (LIT), moderate- (MIT), and high-intensity (HIT)], and specific (skiing/roller skiing) versus unspecific (running and cycling) exercise modes. The training during the 40 gestational weeks (gwk) was analyzed in the first- (Tri-1; gwk 1–12), second- (Tri-2; gwk 13–28), and third trimester (Tri-3; gwk 29–40). The 61 weeks postpartum period was analyzed in (PP1; weeks 1–6, PP2; weeks 7–12, PP3; weeks 13–18, and PP4; weeks 19–24), followed by the general preparation- (GP; weeks 25–44), specific preparation- (SP; weeks 45–53) and the competition period (CP; weeks 54–61).

Pre-pregnancy values are defined as the average training volume in the 5 years before pregnancy during each of the annual training phases, as previously described in detail (Solli et al., 2017).

### Interviews

To gather additional information, ensure compliance with the training diary commentaries, and verify the training intensity of different training sessions, semi-structured interviews were conducted regularly during the data-analysis phase of this study.

### Statistical Analyses

All data from the investigated periods are presented as mean ± standard deviation(median). Training time/sessions were divided by duration (days) of the specific phase and multiplied by seven to determine the weekly time and frequency. All analyses were carried out in Microsoft Office Excel 2016 (Microsoft, Redmond, WA, United States).

## Results

### Physiological and Anthropometric Measurements

Changes in physiological and anthropometric parameters during pregnancy and postpartum are presented in **Table 1**. *V*O_2_ at the estimated lactate threshold (LT) decreased to 95/94% of absolute/body-mass-normalized pre-pregnancy values in Tri-1, 93/89% in Tri-2, and then increased to 100/94% in PP2 and further to 103/98% in PP3. Lean body mass was 96% of pre-pregnancy value during PP1 and increased to 98% during PP4. Fat-% increased from pre-pregnancy to PP1 and progressively decreased to PP4 and CP. Whole body bone mineral density (BMD; g/cm^2^) decreased to 92% of pre-pregnancy value during PP1 and further to 89% during PP3, and thereafter increased to 95–96% during GP and CP.

### Training During Pregnancy

#### Training Volume

**FIGURE 1:**
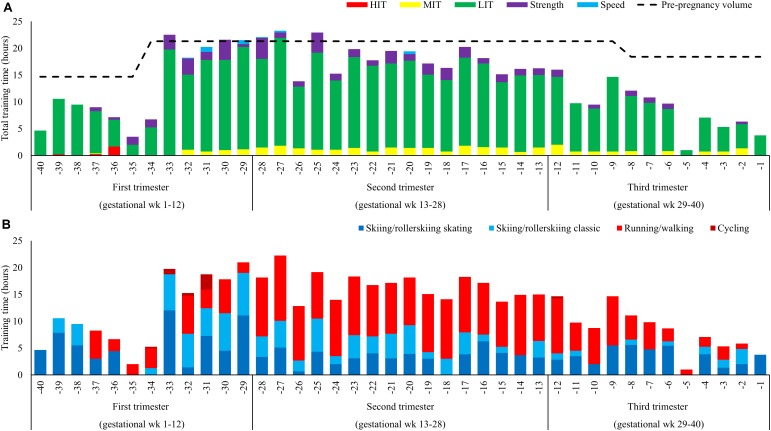
**(A,B)** Training during 40 weeks of pregnancy **(A)** distributed into endurance (low-, moderate-, and high-intensity), strength and speed training and **(B)** distribution into specific (skiing classical or skating) and non-specific activity forms (running and cycling).

Five hundred and fifty-five hours distributed across 316 sessions was performed during the entire pregnancy. This constitutes a weekly average of ∼14 h and 8 sessions. The weekly distribution of training time is presented in **Figure 1A**. Average training volume was 12.9 ± 7.3(10.0) h/week, 18.3 ± 2.9(18.0) h/week, and 8.8 ± 4.4(9.6) h/week during Tri-1, Tri-2, and Tri-3, respectively, corresponding to 79, 86, and 48 % of the same period’s pre-pregnancy training. During Tri-3, the training load was progressively reduced from 12.5 ± 3.3(12.2) h/week (weeks 29–32) to 8.4 ± 5.0(10.3) h/week (weeks 33–36) and 5.6 ± 1.4(5.8) h/week (weeks 37–40).

#### Exercise Modes

The distribution of specific versus unspecific exercise modes is presented in **Figure 1B**. The volume of specific exercise modes was 256 h (101 h skating and 156 h classic) and 249 h was unspecific (244 h running/walking and 5 h cycling). The distribution of specific-/unspecific exercise modes was 74/26, 39/61, and 49/51% during the three trimesters. The participant stopped running during Tri-3.

#### Endurance Training

**FIGURE 2:**
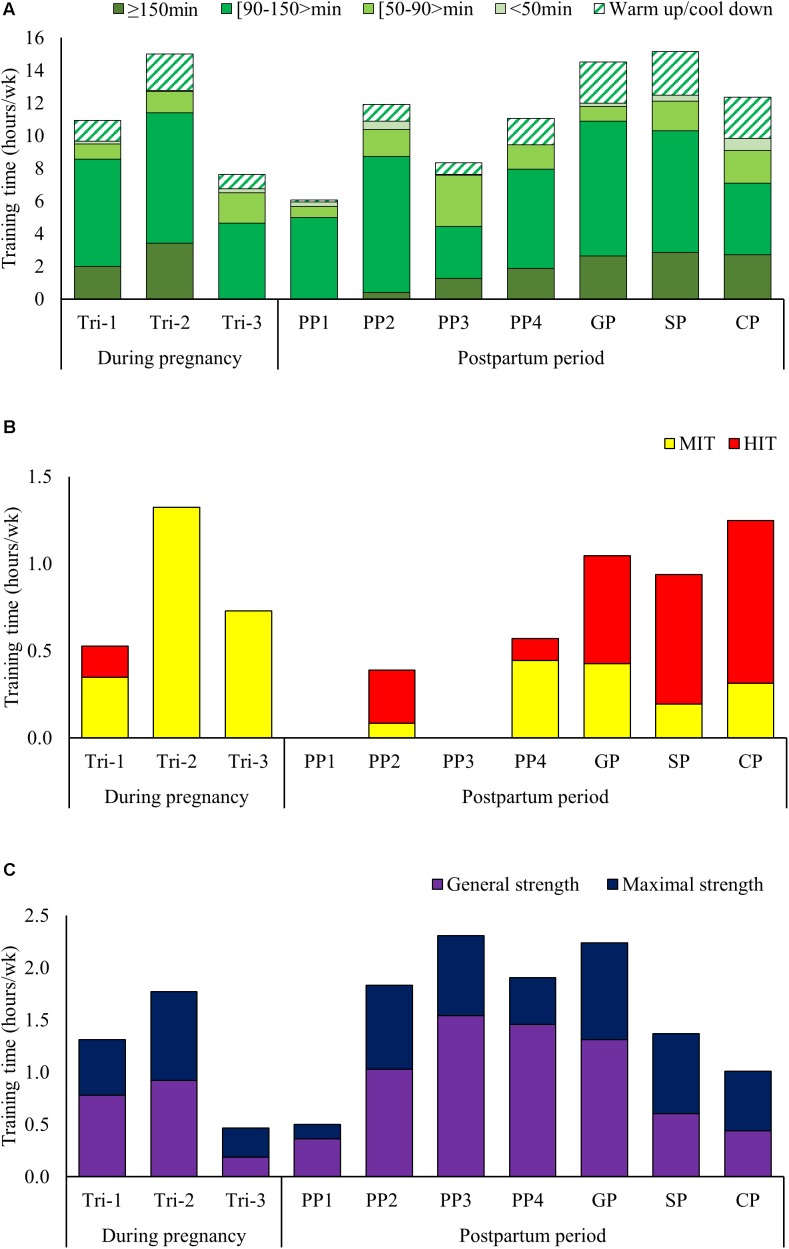
**(A–C)** Low intensity training time categorized after duration **(A)**, distribution of moderate and high intensity training time **(B)** and distribution of general versus heavy strength training time **(C)** across the different phases of pregnancy and postpartum.

Total LIT time was 465 h, distributed as 10.9 ± 6.2(9.9), 15.2 ± 2.3(15.6), and 7.6 ± 3.8(7.9) h/week during Tri-1-3, respectively. Distribution of LIT, MIT, and HIT time during the different phases is presented in **Figures 2A,B**. The number of weekly LIT session’s ≥90 min was 4.3 ± 3.1(3.5), 5.4 ± 1.3(5.5), and 2.7 ± 2.2(2.5) during Tri-1-3, respectively. No session ≥150 min was performed during Tri-3. Total MIT time was 34.1 h distributed across 46 sessions during pregnancy. MIT time was 0.4 ± 0.5(0.0), 1.3 ± 0.4(1.4), and 0.7 ± 0.6(0.8) h/week during Tri-1-3, respectively. Total HIT time during pregnancy was 2.2 h, distributed across three sessions performed during the five initial weeks of Tri-1.

#### Strength and Speed Training

The distribution of strength training time during the different phases is presented in **Figure 2C**. Strength training time was 1.3 ± 1.3(1.1), 1.8 ± 0.9(1.4), and 0.5 ± 0.5(0.3) h/week during Tri-1–3, respectively. The distribution of general-/heavy strength training was 59/41, 52/48, and 40/60% during Tri-1–3, respectively, with gradually more focus on the upper-body and lower focus on trunk and squat exercises. Total amount of speed training during pregnancy was 2.8 h, performed during Tri-1 and 2.

### Training During Postpartum

#### Training Volume

Nine hundred and twenty-three hours distributed across 540 sessions was performed during the 61-week period after delivery until participation in the World championships. The training during this period was interfered by two bone fractions in the sacrum detected during PP3 and PP4.

**FIGURE 3:**
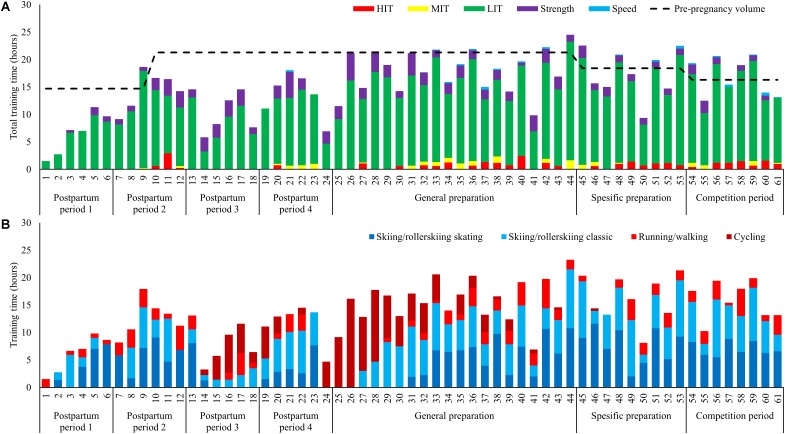
**(A,B)** Training during 61 weeks postpartum **(A)** distributed into endurance (low-, moderate-, and high-intensity), strength and speed training and **(B)** distribution into specific (skiing classical or skating) and non-specific activity forms (running and cycling).

Training time increased progressively from ∼2 to 11 h/week during PP1. The weekly distribution of training time is presented in **Figure 3A**. Average training volume was 6.6 ± 3.8(7.1) h/week during PP1, 14.1 ± 3.4(14.3) h/week during PP2, 10.6 ± 3.8(10.4) h/week during PP3 and 13.6 ± 4.1(14.5) h/week during PP4. The training volume further increased to 18.0 ± 3.9(18.7) h/week during GP (weeks 25–44), 17.6 ± 4.4(17.3) h/week during SP (weeks 45–53) and 16.9 ± 3.5(17.2) h/week during CP (weeks 54–61), corresponding to 85, 95, and 104% of the volume in the same phases pre-pregnancy.

#### Exercise Modes

The distribution of specific versus unspecific exercise modes is presented in **Figure 3B**, with a distribution of specific-/unspecific exercise modes being 85/15, 78/22, 43/56, and 69/31% during PP1-4, respectively. The amount of cycling was 3.3 ± 2.7(3.1), 2.5 ± 2.5(3.2), and 4.5 ± 4.7 (3.4) h/week during PP3, PP4, and GP, respectively. This is 3–5 times higher than pre-pregnancy values during the same phases.

#### Endurance Training

Low intensity training time was distributed as 6.1 ± 3.3(6.8), 11.9 ± 3.4(10.6), 8.3 ± 3.8(8.0), and 11.1 ± 3.3(12.1) h/week during PP1-4, respectively, with the number of LIT session’s ≥90 min being 2.8 ± 1.9(3.5), 4.8 ± 1.8(4.4), 2.2 ± 1.5(2.1), and 4.0 ± 1.1(4.0) session/week. All of the endurance training time during PP1 consisted of LIT, and the first LIT session ≥150 min was performed during PP2. MIT and HIT was re-introduced during PP2, but withdrawn during PP3 due to bone fraction, then re-introduced during PP4, but then withdrawn again with the second fraction until it was re-introduced on a permanent basis from week 30. The amount of MIT/HIT time was 0.4/0.6, 0.2/0.7, and 0.3/0.9 h/week during GP, SP, and CP.

#### Strength and Speed Training

The first general strength training session was performed in week 3 and the first heavy strength session was performed during week 5. Strength training volume was 0.5 ± 0.6(0.3), 1.8 ± 1.0(1.6), 2.3 ± 0.8(2.5), and 1.9 ± 1.8(2.2) h/week during PP1-4, respectively, with the distribution of general-/heavy strength training being 72/28, 56/44, 67/33, and 77/23%. The amount of strength training was 2.2 ± 1.1(2.1), 1.4 ± 0.4(1.3), and 1.0 ± 0.8(1.0) h/week during GP, SP, and CP, respectively, corresponding to 109, 114, and 126% of pre-pregnancy values. Speed training was introduced in week 33 and thereafter employed regularly 0.1–0.2 h/week.

## Discussion

In this case-study, the main aim was to investigate training characteristics, physiological capacity and body composition of the world’s most successful XC-skier during the entire pregnancy, and the 61 weeks postpartum. During the first and second trimester, the average training volume was ∼80–85% of pre-pregnancy values, but then progressively decreased to ∼50% during the third trimester where training was gradually reduced throughout. While LIT and MIT was performed throughout pregnancy, no HIT was performed after gestational week 5 and strength training was progressively modified. In postpartum, the participant had two setbacks caused by fractures in the sacrum in PP3 and PP4. However, by reducing the overall training load, slower progression and utilization of alternative exercise modes, the participant had a successful training development and return to competition.

### Training During Pregnancy

The participant’s average training volume during pregnancy was 14 h/week, which included 79, 86, and 49% of pre-pregnancy volumes during the first, second, and third trimester, respectively. The absolute volume done by our athlete is much higher than, for example, the average 8.4 h/week suggested for a rapid return to competitive sport, without jeopardizing the fetus health (Kardel, 2005). However, the relative values are more in line with marathon runners reporting average running volumes of 40 and 107 km/week during pregnancy, corresponding to ∼40 and ∼70% of their pre-pregnancy volume (Potteiger et al., 1993; Bailey et al., 1998). Still, this study provides the highest training volume during pregnancy ever reported in the literature. This could partly be explained by the varied exercise modes utilized in XC-skiing, e.g., allowing to reduce the mechanical stress compared to running, and partly by the high pre-pregnancy training volume and the high relative amounts during pregnancy.

The amount of HIT was substantially reduced compared to pre-pregnancy, with no HIT sessions performed after gestational week 5. This is in line with a previous study reporting that an exercise intensity >90% of maximal maternal heart rate may reduce the blood flow to the uterus and result in fetal bradycardia (Salvesen et al., 2012). In contrast, MIT was performed throughout the entire pregnancy, and with higher amounts than pre-pregnancy during the second trimester. Likely, this was an effective substitute for the reduced HIT in order to maintain performance level as high as possible during pregnancy.

During the second trimester, the athlete joined a 14-day training camp at altitude (1800 meter above sea level), and endured a training volume of ∼22 h/week (i.e., 85% of pre-pregnancy altitude volumes) (Solli et al., 2017). However, only LIT and MIT was performed, and the camp was performed without any abnormalities beyond the ability to keep the same training speed as before pregnancy. Because of a possible reduction in the fetus oxygen saturation, intensive training at altitude is not recommended during pregnancy (Entin and Coffin, 2004). However, since XC-skiers normally has a lower proportion of HIT during altitude (Sandbakk and Holmberg, 2017), the participant could follow approximately the same training plan as her team mates.

The relative utilization of exercise modes was approximately the same compared to pre-pregnancy during the first and second trimester, but the amount of running was reduced during the third trimester. Specifically, our participant reported increased soreness in the muscles around her hip after running sessions during the third trimester, and stopped running approximately 6 weeks before giving birth. This is in line with previous studies (Tenforde et al., 2015), and is likely caused by the increased body weight and the changes in biomechanical stress as the gravity center alters during pregnancy.

Both general and heavy strength training was performed throughout the whole pregnancy, but with a clear volume-reduction during the third trimester. In addition, the strength training program was gradually modified, e.g., with more focus on upper-body exercises and less focus on abdominal muscles and squats. This is in line with previous reports in recreational athletes where low-resistance strength training has shown no negative effects for the fetus (Avery et al., 1999), and no negative effect on the pelvic floor was reported in cross-fit athletes performing heavy lifts during pregnancy (Middlekauff et al., 2016). In any case, the modification in strength training might have contributed to the small decline (4%) in lean body mass during pregnancy. However, more research concerning both the load and types of exercise (e.g., upper vs. lower-body muscles), and the subsequent effects on maintenance of muscle mass, is needed to give more accurate recommendations to elite athletes.

### Training During Postpartum

The participant had a quick return to training, and progressively increased training volume to 11 h/week during PP1. In PP2, this was further increased to 19 h/week and MIT and HIT was reintroduced. Strength training was included from week 3, and progressively increased to 2 h/week during PP2. However, coinciding this rapid increase in training load a fracture in the sacrum was detected during PP3. This subsequently led to a reduction in the training volume, followed by a new progression in the training during PP4, until a new fracture on the other side of the sacrum was detected. Although similar progression of training load during postpartum, without injuries, has previously been reported in a marathon runner (Potteiger et al., 1993), it is likely that these injuries occurred because of a too rapid progression. In this connection, a decrease in the BMD after delivery and further lowering to PP3 was observed. The reason for this might be that the fetal skeleton requires a substantial transfer of calcium during Tri-3 and, in addition, loss of calcium in breast milk (Sanz-Salvador et al., 2015). Currently, the mechanisms behind calcium transfer and bone turnover during pregnancy and lactating are only partly understood and there is a lack of knowledge considering the effect of exercise on these factors. However, it is likely that pregnancy is a vulnerable period for the mother’s bones that especially elite athletes should be aware of.

After the second fraction, the amount of MIT and HIT was reduced and running was replaced with cycling during the following 7 weeks. At the end of GP, MIT, and HIT was reintroduced on a permanent basis and the participant immediately responded positively and gained a substantial performance improvement. In the final weeks of GP she participated at her first altitude camp after delivery, and experienced that the training during altitude was easier than pre-pregnancy. In SP the training volumes was back at pre-pregnancy levels. During CP, she followed the same tapering pattern as before pregnancy (Solli et al., 2017), but with more day-to-day adjustments of training due to the new obligations with a newborn child and more pronounced reduction of her training during the two final weeks before the successful World Championship in Lahti 2017.

### Ethical Considerations and Practical Recommendations

The following factors should be considered when implementing the findings of this study to other athletes/sports:

• *Maternal age* > 35 years is associated with several pregnancy complications (Lean et al., 2017).
• *A larger number of pregnancies and deliveries* could likely influence risks associated pregnancy.
• The *pre-pregnancy training volume* of the participant was high due to progressive increase in training volume over many years (Solli et al., 2017).
• The *characteristics of XC- skiing where* several exercise modes is utilized in training (i.e., skiing, roller-skiing, running, and cycling), which makes utilization of alternative exercise modes easier.
• Requirements of *hydration and nutrition* increases during pregnancy (Bo et al., 2016), an aspect carefully taken care of in this case.
• The *supporting network* closely supervised our participant, which is not necessarily the case for athletes at lower performance levels.

## Conclusion

This study provides unique data of the training characteristics, physiological capacity and body composition for the world’s most successful XC-skier during pregnancy and the year postpartum. Our data indicates that high level XC-skiers can tolerate high training loads during pregnancy. However, elimination of HIT, modified strength training and a gradually reduced training load during the third trimester seems to be required. In postpartum, the setbacks in our participant’s training, due to fractures in the sacrum, was likely caused by a too rapid progression of training. However, by reducing the overall training load, followed by slower progression and utilization of alternative exercise modes, the participant had a successful return to competitions and managed to win four gold medals in the subsequent World Championship.

## Author Contributions

GS performed data collection and performed data and statistical analysis. GS and ØS designed the study, contributed to interpretation of the results, wrote the draft manuscript, and contributed to the final manuscript.

## Conflict of Interest Statement

The authors declare that the research was conducted in the absence of any commercial or financial relationships that could be construed as a potential conflict of interest.
